# Patient-reported outcomes labeling for oncology drugs: Multidisciplinary perspectives on current status and future directions

**DOI:** 10.3389/fphar.2022.1031992

**Published:** 2022-10-17

**Authors:** David Cella, Chieh-I Chen, Ruben G. W. Quek, Ainhoa Uribarren, Matthew Reaney, Vera Mastey, Deborah Collyar, Olivier Chassany

**Affiliations:** ^1^ Northwestern University Feinberg School of Medicine, Chicago, IL, United States; ^2^ Regeneron Pharmaceuticals Inc., Tarrytown, NY, United States; ^3^ IQVIA, Reading, United Kingdom; ^4^ Patient Advocates in Research (PAIR), Danville, CA, United States; ^5^ Health Economics Clinical Trial Unit (URC-ECO), Hôpital Hotel-Dieu, Paris, France; ^6^ Patient-Reported Outcomes Unit (PROQOL), Université de Paris, Inserm, Paris, France

**Keywords:** quality of life, patient reported outcome instruments, patient care, PRO labeling, oncology

## Abstract

**Introduction:** Regulatory agencies encourage the incorporation of the patient voices throughout clinical drug development. Patient-Reported Outcomes (PROs) offer one way of doing this and their use has markedly increased in many therapeutic areas, particularly oncology, in recent years. However, few oncology drug labels include PRO data and those which do, offer little consistency.

**Objective:** To provide multidisciplinary perspectives (patient, pharmaceutical industry, PRO researcher, regulatory expert) on PRO data in oncology drug labels.

**Methods:** PRO data in the labels of drugs approved by the Food and Drug Administration (FDA) and European Medicines Agency (EMA) for oncology indications between 2010 and 2020 were critically reviewed by authors who provided their insights on the advantages and disadvantages/gaps.

**Results:** Forty-six oncology drugs included PRO data in their labels. Differences were observed between FDA and EMA PRO labeling (e.g., PRO concept, use of tables and graphs to display PROs or reference to clinical meaningfulness). In providing their perspectives on the number and nature of PROs in labels, authors noted limitations including: the low proportion of oncology drugs with PRO labeling, limited PRO information in labels, lack of patient-friendly language, and potential bias towards positive outcomes. Lack of consistency within- and between-agencies was noted.

**Conclusion:** Despite regulatory agencies’ commitment to incorporate patient voices in regulatory decisions, availability of PRO information is limited in oncology drug labels. While several PRO guidance documents are available from regulatory and Health Technology Assessment agencies, harmonization of PRO guidance for labeling inclusion around the world is needed to better inform prescribers and consequently their patients in the process of shared medical decisions.

## 1 Introduction

In the last decade, the weight of patient voices and the release of several official guidelines from the US Food and Drug Administration (U.S. Department of Health and Human Services Food and Drug Administration, 2019) (FDA) and European Medicines Agency (EMA Committee for Medicinal Products for Human Use, 2019) (EMA), strongly encourage sponsors to include Patient-Reported Outcomes (PROs) in clinical trials in many therapeutic areas, particularly in oncology. Inclusion of PROs in the clinical development of new drugs (Bottomley et al., 2019) has long been advocated by patients and healthcare providers to provide a patient-centered holistic understanding of the potential benefits and/or concerns associated with new drugs.

Over the past 10–15 years, systematic consideration and formal incorporation of PRO data into regulatory (U.S. Department of Health and Human Services Food and Drug Administration, 2019; EMA Committee for Medicinal Products for Human Use, 2019; U.S. Department, 2009; FDA, 2017; U.S. Department of Health and Human Services. Food and Drug Administration. Oncology Center of Excellence, 2018) and health technology (Ara and Wailoo, 2011; HAS. Transparency Committee doctrine, 2019; Böhme et al., 2021; Scope et al., 2022) agencies considerations and guidance has increased. The FDA (CDER, 2019) and EMA (EMA, 2019) have expressed interest in the patient perspective in regulatory decision-making in oncology. Specifically, FDA’s Center for Drug Evaluation and Research Patient-Focused Drug Development task force drafted four guidance documents to provide a framework and enhance the incorporation of patient voices in medical drug development and regulatory decision making (CDER, 2019). EMA has published their future regulatory science strategy (EMA, 2019) which similarly highlights opportunities to incorporate PROs and patient preferences into drug development and risk-benefit assessment (EMA, 2019). Both agencies have also published oncology-specific PRO guidances (U.S. Department of Health and Human Services Food and Drug Administration, 2019; EMA Committee for Medicinal Products for Human Use, 2019). These guidance documents highlight a role for PROs to inform benefit-risk appraisal for new drugs, and describe the potential inclusion of PRO data in drug labeling (i.e., US Prescribing Information and EU summary of product characteristics) where the evidence supports it. However, while multiple PRO guidelines are available from regulatory and Health Technology Assessment agencies, they are not always consistent. With potential differing regulatory approval standards across regions and countries, guidance surrounding PRO may naturally differ. In addition, Health Technology Assessment agencies and regulatory agencies may have different objectives regarding their assessment of PRO evidence. Nevertheless, a more harmonized approach across regions and agencies is desirable to maximize the utility of data and to ensure that drug development companies have an unambiguous direction to follow during protocol development, endpoint positioning, and pre-specified analyses. Some innovative oncology treatments extend life expectancy; PRO data may provide patients and physicians additional information and context about benefit-risk profile in those settings where several treatment options are available offering similar survival benefit.

PROs are also becoming more important in payer decisions to assess the full value and added value of new therapies. Examples include guidance from the European Network for Health Technology Assessment (EUnetHTA. Guideline, 2013), European Society for Medical Oncology (Dafni et al., 2017) and Institute for Clinical and Economic Review (Institute for Clinical and Economic Review, 2020) which highlight the potential role of PROs in determining the full value of therapies. The Institute for Clinical and Economic Review Value Assessment Framework (Institute for Clinical and Economic Review, 2020) specifies that if PROs have not been collected in the manufacturer’s clinical development program, the Institute for Clinical and Economic Review conducts a comprehensive literature review to identify observational studies providing this information (Institute for Clinical and Economic Review, 2020).

In addition to other platforms such as social media, medical literature, Project Patient Voice (PPV) or scientific congresses, the drug label is a potential avenue to communicate the patient experience to physicians and patients to inform prescribing decisions, but few oncology drugs have been granted PRO labeling (ERG, 2019; Gnanasakthy et al., 2019). Such patient experience information in the drug label could be leveraged to develop lay summaries published by the EMA; similar initiatives are underway in the US and in Canada (Barnes and Patrick, 2019). Several hurdles preclude PRO inclusion in labeling, including large amounts of missing data, concern about bias introduced by open label or uncontrolled trial designs, lack of sufficient evidence for the validity of the PRO instrument, and failure to include PROs in the endpoint hierarchy for statistical testing (U.S. Department, 2009; Gnanasakthy et al., 2019; Basch et al., 2015). Furthermore, the label is restricted by space, with limited flexibility to allow for full data description (FDA, 2022).

The current study aims to:

1) Review and appraise PRO data in both FDA and EMA labels of oncology drugs approved between 2010 and 2020; and
2) Conduct an assessment of the PRO data in FDA and EMA labels of oncology drugs. Assessment focused on the (in)consistency, relevance and clarity of PRO data across labels, advantages and disadvantages of PRO data being included in drug labeling, and opportunities for improvement. Each author provided an independent assessment, covering patient, PRO researcher, regulatory expert, and pharmaceutical industry perspectives.

## 2 Materials and methods

### 2.1 Identification of patient-reported outcomes labeling in oncology

An initial list of oncology drugs with PRO labeling was obtained from PROLABELS™ (Mapi Research Trust), a database containing PRO data from the US and EU labels published by the FDA and EMA, respectively, where at least one PRO domain and/or instrument is mentioned in the efficacy or safety sections of the main documents (Mapi Research Trust, 2022). Drug approvals, revisions and withdrawals are reviewed daily and updated on the PROLABELS™ database within 1 month (Mapi Research Trust, 2022).

The search was conducted in December 2020. Filters included “neoplasms” for therapeutic area and “PRO” for type of outcome assessment. The FDA US Prescribing Information and the EMA EU summary of product characteristics of oncology drugs approved between January 2010 and December 2020 were reviewed to characterize the PRO labeling in terms of PRO concept (health-related quality of life, patient preference, symptom, functioning, health status), instrument used to assess the PRO, and the format (text and/or table or graphic) and text of the PRO labeling. Irrespective of the number of PRO concepts or endpoints included in the label, a single PRO labeling per drug per indication was used in our metrics. Details of the study design, the endpoint hierarchy and the PRO-related analyses were also captured when available. Drugs approved for oncologic diseases that are considered benign (e.g., leiomyoma) were excluded; overall, four FDA and one EMA labels were excluded. In addition, the label of generics used in oncology and approved during this period were also excluded to avoid double counting. The label of drugs taken off the market were also excluded but biosimilars were not.

### 2.2 Appraisal of patient-reported outcomes labeling

The PRO data in the labels were reviewed by all authors and critically appraised from their perspectives which included that of patients (*n* = 1; Collyar), PRO researchers (*n* = 4; Chassany, Cella, Reaney, Uribarren), pharmaceutical industry (*n* = 3; Chen, Mastey, Quek), and regulatory experts (*n* = 1; Chassany). Authors were asked to answer six questions from their perspective(s) (Supplementary Table S1); each author could assess the label from more than one stakeholder standpoint based on their backgrounds. The questions were designed by one of the authors and approved by all authors. Authors provided their insights on the advantages and disadvantages/gaps of PRO data being included in drug labeling (question 1); and (in)consistency of PRO data across labels (question 2). They were also asked how informative and clear PRO data in labels are (question 3), and about the relevance of the PROs in the labels to them and whether PRO data from labels are used differently compared to PRO data from scientific publications (question 4). Finally, they were asked about improvements they would like to see in PRO labeling (question 5) and other avenues that may be appropriate for communication and presentation of PRO data (question 6). Each author answered the questions independently.

## 3 Results

### 3.1 Oncology drugs with patient-reported outcomes labeling

Between 2010 and 2020, out of 169 (FDA: *n* = 108; EMA: *n* = 139) drugs approved in one or more oncology indications, 46 drugs included PRO data for at least one oncology indication in the FDA [*n* = 9/108 (8.3%) drugs in nine indications] or EMA [*n* = 42/139 (30.2%) drugs in 53 indications] labels. Five drugs included PRO data in both, FDA and EMA labels (Table 1; Supplementary Table S2). Among the oncology drugs with PRO labeling approved by EMA between 2010 and 2020, 77% (*n* = 41/53) of them were approved from 2015 onwards. All FDA oncology drugs with PRO labeling were approved from 2014 onwards (data not shown).

**TABLE 1:** Overview of EMA and FDA PRO labeling in oncology.

	FDA	EMA
Number of oncology drugs approved in 2010–2020	108	139
Number of drugs with PRO labeling, n (%)	9^a^ (8.3)	42^a^ (30.2)
Number of indications with PRO labeling	9	53
PRO concept, n (%)	9	53
HRQoL	0 (0.0)	39 (73.6)
Functioning	0 (0.0)	11 (20.7)
Symptoms	6 (66.7.3)	17 (32.1)
Pain	5 (55.6)	12 (22.6)
Fatigue	1 (11.1)	1 (1.9)
Dyspnea^b^	2 (22.2)	4 (7.6)
Cough	1 (11.1)	3 (5.7)
Diarrhea	0 (0.0)	1 (1.8)
Health utility index	0 (0.0)	11 (20.7)
Patient preference	3 (33.3)	0 (0.0)
Patient-reported use of rescue treatment	2 (22.2)	2 (3.8)
Studies providing PRO data in label	9	57
Double blinded^c^, n (%)	4 (44.4)	27 (47.4)
Open label^c^, n (%)	4 (44.4)	28 (49.1)
Single arm^c^, n (%)	0 (0.0)	1 (1.7)
Unclear^c^, n (%)	1 (11.1)	1 (1.7)
Endpoints and analyses^c^	18	63
Primary endpoint, n (%)	2 (22.2)	0 (0.0)
Secondary endpoint, n (%)	5 (55.5)	46 (96.8)
*Post-hoc* analysis^c^, n (%)	0 (0.0)	1 (1.9)
Secondary and exploratory endpoint, n (%)	1 (11.1)	1 (1.9)
Exploratory endpoints, n (%)	1 (11.1)	7 (13.2)

^a^ Of these, 5 (9.3%) received PRO labeling by both regulatory agencies.

^b^ Also referred to as shortness of breath in several labels.

^c^ Among all studies; all other percentages are over the total number of drugs with PRO labeling.

^d^ The two instruments most commonly cited in EMA labels.

^e^ The two type of instruments most commonly cited in FDA labels.

^f^ The diaries included the modified Myelofibrosis Symptom Assessment Form v2.0 diary [fedranitib (FDA), ruxolitinib (FDA)], an electronic diary to capture rescue medication, and severity and frequency of diarrhea and flushing symptoms [lanreotide (FDA)], a diary to capture bowel movements [telotristat ethyl (FDA, EMA)].

^g^ The labels (pertuzumab, rituximab, trastuzumab) do not provide any details on the preference questionnaire used in the trials. Abbreviations: EORTC QLQ-C30, European organisation for research and treatment of cancer quality of life questionnaire core; EQ-5D, EuroQoL 5 dimension; PRO, patient-reported outcome.

PRO concepts in the FDA labeling (*n* = 9) included symptoms in 6 [66.7% with three referring to a single symptom (pain: *n* = 2; short of breath: *n* = 1)], and patient preference in 3 (33.3%) labels (Table 1). PRO concepts in EMA labeling (*n* = 53) included health-related quality of life in 39 (73.6%), functioning in 11 (20.7%), symptoms in 17 (32.1%) and health utility in 11 (20.7%) EU labels (Table 1). The focus on symptoms at FDA is in line with their PRO Guidance (U.S. Department, 2009) which emphasizes the intended and direct effect of treatment (sign/symptom improvement), while the focus on health-related quality of life at EMA is in line with their guidance (EMA Committee for Medicinal Products for Human Use, 2019) emphasizing the relevance of “consequences for the daily life and social functioning” of these core signs and symptoms. The most cited PRO instruments differed largely between FDA and EMA labels, i.e., European Organisation For Research And Treatment Of Cancer core Quality of Life Questionnaire (EORTC QLQ-C30) and the EuroQoL-5 dimensions (EQ-5D) for EMA vs. patient preference questionnaires for FDA (Supplementary Table S3). These differences are unlikely due to differences in the data submitted to these agencies but probably to differing evidence-related standards between FDA and EMA. These differences were also observed for those oncology drugs with PROs in both, the FDA and EMA, label (e.g., pain progression in the FDA abiraterone label for chemotherapy-naïve metastatic castration-resistant prostate cancer vs. pain progression and Functional Assessment of Cancer Therapy—Prostate [FACT-P] total score in its EU label).

In terms of study designs leading to the PRO labeling, 4 (44.4% of nine studies) and 28 (49.1% of 57 studies) were open-label for FDA and EMA approvals, respectively and one EMA approval cited a single arm study (Table 1). Whilst this reflects the oncology trial landscape, with oncology trials more likely to be single arm, open label, and nonrandomized compared to non-oncology trials (Hirsch et al., 2013)—primarily for practical reasons—this is inconsistent with the FDA and EMA stated concerns about interpretability of PRO data when studies are not double-blinded (EMA Committee for Medicinal Products for Human Use, 2019; U.S. Department, 2009).

Although guidance from FDA (U.S. Department, 2009; CDER, 2017) and EMA (EMA. Guideline, 2016) suggests that alpha-controlled endpoints are prioritized for labeling, some labels included text suggesting that the PRO data was derived from exploratory analyses (as a function of being completed *post-hoc* or not being alpha-controlled). It is uncommon for labels to report the endpoint hierarchy or whether the analysis plan was specified with alpha allocation. Based on the label information and further research in the Assessment Reports of EMA approvals, and the clinical and/or Statistical Reviews of FDA approvals, 2 (22.2%) and 8 (15.1%) of the FDA and EMA PRO labelings, respectively, related either exclusively or partly on exploratory endpoint, and 1 (1.9%) of the EMA labelings to *post-hoc* analyses of a secondary endpoint (Table 1). Two of the FDA PRO labelings were based on primary endpoints (patient preference in both cases).

Most FDA (78%) and EMA (94%) PRO labelings were communicated solely as text provided in section 14 (“clinical efficacy”) of the FDA label and section 5.1 (“pharmacodynamic properties”) of the EMA label. Of the FDA and EMA labels with PRO labeling, only 2 (22.2%) and 3 (5.7%), respectively had a table, and 2 (22.2%) and 0 a graph. The two FDA labels with tables and/or graphs included both (Table 2).

**TABLE 2:** Format used to communicate the PRO labeling.

	FDA (%)	EMA (%)
Text	9	53
Descriptive with no estimates or *p*-values, n (%)	2 (22.2)	37 (69.7)
Descriptive with no estimates or *p*-values but with reference to statistical significance, n (%)	0 (0.0)	7^a^ (15.1)
Numerical values, n (%)	7 (77.8)	24 (45.3)
*p*-values, n (%)	4 (44.4)	15 (28.3)
Reference to whether results were statistically significant or not, n (%)	1 (11.1)	13^b^ (35.7)
Reference to clinically meaningfulness, n (%)	0 (0.0)	17 (32.1)
Responder definition, n (%)	2 (22.2)	13 (24.5)
Table, n (%)	2 (22.2)	3 (5.4)
Graph, n (%)	2 (22.2)	0 (0.0)
Bar chart, n (%)	2 (100.0)	0 (0.0)
Waterfall, n (%)	2 (100.0)	0 (0.0)

^a^ In addition, three claims reported that the findings were significant but did not specific if they were significant from a statistical point of view.

^b^ In addition, nine claims reported that the findings were significant but did not specific if they were significant from a statistical point of view.

**FIGURE 1:**
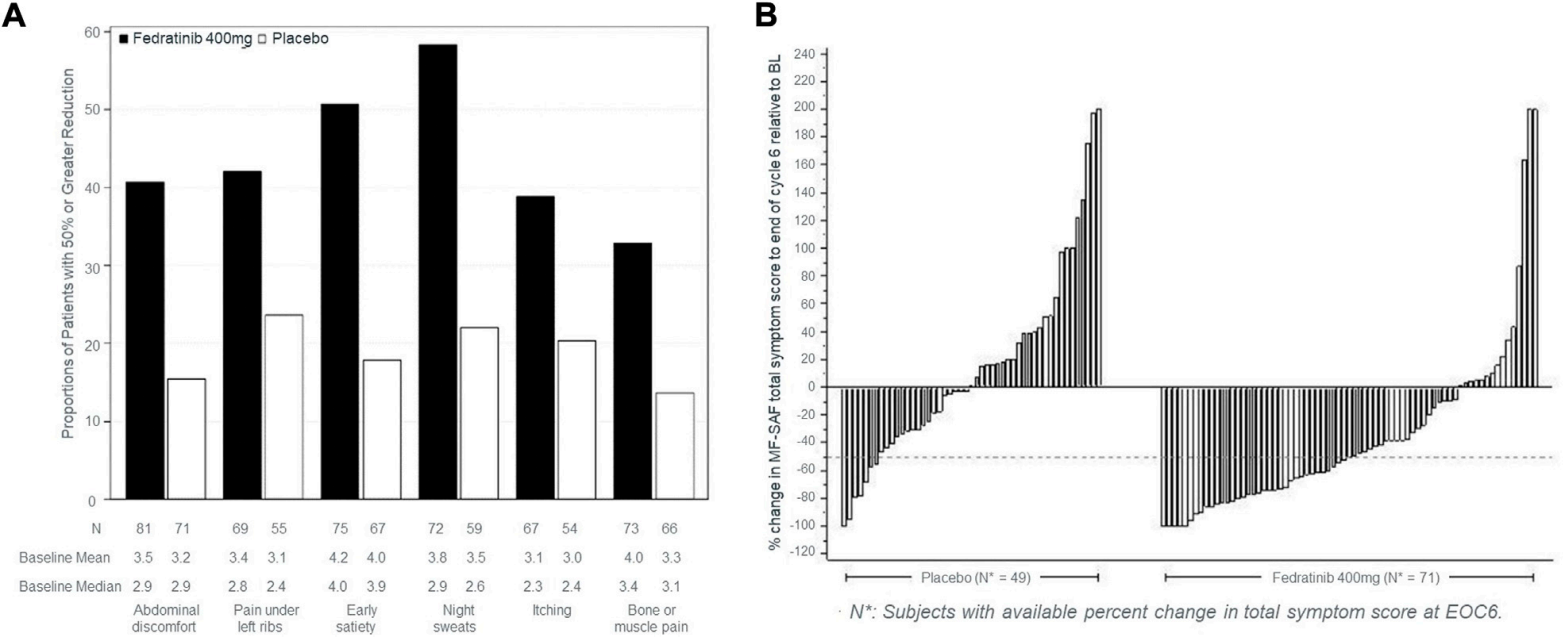
Type of graphs used in FDA labels to commnicate PROs. **(A)** Proportion of patients achieving ≥50% reduction in individual symptom scores at the end of cycle six with non-zero baseline scores. **(B)** Percent change from baseline in total symptom score at the end of cycle six for each patient in the phase three study, JAKARTA. The graphs in **(A,B)** were taken from the US product insert of fedranitib (Inrebic®). These two types of graphs are also included in the ruxolitinib (Jakafi®) US product insert.

Among FDA labels, tables were used to provide the proportion of patients who improved (fedranitib, ruxolitinib). In EMA labels, tables provided average score at each visit (padeliporfin), change from baseline (mixed model repeated measures for osimertinib), proportion of patients who improved (afatinib) and median time to deterioration (afatinib). Regarding graphs, FDA labels for two drugs (fedranitib; ruxolitinib) each had a bar chart and a waterfall plot (Figure 1).

Among the nine drugs with PRO data in their FDA labels, 2 (22.2%) were descriptive with no numerical data, *p*-value or reference to statistical significance or clinical meaningfulness of the data. *p*-values were reported in four labels (44.4%; Table 2). Two (22.2%) included the threshold used in the responder definition to define within-person meaningful changes and reported the proportion of patients with meaningful changes (all standalone analyses; e.g., “≥50% reduction in Total Symptom Score in 40% in the INREBIC group and 9% in the placebo group” in the fedratinib FDA label).

Among the 53 drugs with PROs in their EU label, PROs were descriptive for 37 (69.8%) of them with no numerical data, or *p*-values, however seven of these EU labels mentioned whether the outcomes were statistically significant or not. Overall, *p*-values were reported for 15 (28.3%) drugs. In addition, 13 (24.5%) reported the threshold used in the responder definition, 2 (3.8%) the proportion of patients with meaningful changes and 17 (32.1%) referred to the clinical meaningfulness of the mean change data [with 10 (e.g., brentuximab and obinutuzumab) of these labels not reporting the threshold; Table 2].

### 3.2 Multidisciplinary perspectives

#### 3.2.1 Advantages and disadvantages or gaps of patient-reported outcomes data in labeling

Each author, from their collective perspectives, identified between four and nine advantages to having PRO data included in drug labels. The authors generally felt that PRO data inferred meaningfulness to patients (i.e., it was measuring a concept of interest), providing a more complete perspective of the benefit-risk profile of therapies for patients (see Table 3). Communicating this PRO data through the label reflects a willingness of regulators to reflect the patient perspective in approval documents, encourages consideration by payers, and allows the information to be proactively shared with clinicians and patients; all things perceived as an advantage by the pharmaceutical industry and/or PRO researchers. Having data in label also infers high data quality which can both be used to supplement clinician assessments of treatments and inform use of those treatments in clinical practice—seen as an advantage by the patients (Table 3). The patients, PRO researchers and pharmaceutical industry also discussed the appreciation that different labels reflected different PRO data. While it may be appropriate to consider a core set of outcomes for capturing the patient perspective of oncology medication (U.S. Department of Health and Human Services Food and Drug Administration, 2019), overall PRO strategies must also be considered in light of the specific population, treatment, and study design for a drug development program, with additional non-core outcomes important in certain situations. The wide-ranging PRO label claims (by concept and instrument) was seen as an advantage.

**TABLE 3:** Advantages and gaps of PRO data in drug labeling from different perspectives.

	Patient	Pharmaceutical industry	PRO researcher	Regulatory expert
Advantages of PROs in labeling				
Refer to endpoints that are meaningful to patients (i.e., present patients with data that is relevant to them)	X		X	X
Provide a more holistic perspective of benefit risk drug profile (i.e., incorporate the patient perspective into the appraisal of the drug)		X	X	X
Reflect the humanistic value of drugs to payers (i.e., patient-perceived value on outcomes which are not core to defining safety/efficacy)		X		
Reflect willingness of regulators to capture information deemed important to patients in the label (i.e., patient-focused drug development)			X	
Supplement clinician’s assessments with information directly from patients	X			
Informs future use of treatments (i.e., PRO data in the label can be used in treatment decision-making)	X			
Can be used as promotional material (i.e., can be used in direct communication to clinicians and patients)		X	X	
Heterogeneity of PROs in labeling reflects heterogeneity of patient experience within and across diseases (i.e., disease- and treatment-specific strategies encouraged as relevant)	X	X	X	
Gaps of PROS in labeling				
PROs in labeling do not fully capture patients perspective (i.e., often reflect only few of the collected PRO data and thus are insufficiently comprehensive to capture all patient relevant information)^a^	X	X		
Low number of drugs with PRO labeling (i.e., only few drugs with PRO data have these data in the label)	X			
There does not seem to be clear criteria from regulatory bodies for inclusion of PROs in labeling (i.e., apparent inconsistency in labeling decision-making)			X	X
Underrepresentation of certain study designs (e.g., open-label) in PRO claims		X		
Lack of acknowledgement of investment (i.e., cost of developing and/or utilizing PROs does not guarantee use in labeling)			X	
Heterogeneity in labeling across drugs makes it difficult to do meta-comparisons across treatments	X	X	X	
Inconsistencies in core concepts, PROs and/or analyses across studies [i.e., no common data element (CDE) definitions for consistency]	X		X	
Inconsistencies across labels render interpretation of the PRO results difficult for regulators and clinicians				X

The table summarizes the authors responses to question number one of Supplementary Table S1, i.e., “What do you see as the advantages and disadvantages of PRO data being included in drug labeling?” A given author could provide their perspective from different stakeholders.

^a^ Because of limited amount of collected PRO data being included in the labeling and because not all PRO tools are appropriate.

Each author also identified disadvantages or gaps in current PRO labels. These differed across authors. Incomplete PRO data included in labeling may create a false sense of relevance or importance while implying irrelevance for that which is not included, according to patients and the pharmaceutical industry. That is, where multiple PROs were collected to provide a holistic picture, but where only some of that PRO data is represented in labeling, the overarching impact of treatment on areas important to patients may be missing. While the criteria for defining a PRO measure as “fit for purpose” to support regulatory labeling are well-established (EMA Committee for Medicinal Products for Human Use, 2019), there is a lack of clarity in some cases as to why some PRO label claims have been granted while others have not. This was highlighted from PRO researchers and regulatory expert standpoints.

The lack of consistency in label language was also highlighted and authors were explicitly asked about the impact of this inconsistency across drug labels. Authors acknowledged that different indications, populations and treatment lines may necessitate different approaches to measure outcomes that are important to patients. They also acknowledged the large number of PRO instruments available to oncology researchers. Inclusion of broad concepts in labels was considered as a reflection of the heterogeneity of patient experience within and across diseases, and a sign of regulatory willingness to capture the diversity of relevant patient experiences (Table 3). The authors also acknowledged that different stakeholders have an interest in different concepts. However, this creates difficulties to compare PROs across alternative treatments when conducting (network) meta-analyses; authors highlighted the need to identify core outcome sets or common data elements to assess across studies for PRO instruments (Table 3). Consistency on analytical approaches is also needed. From a regulatory expert perspective, inconsistency limits the possibility to train regulators and clinicians in interpreting PRO results (Table 3). Further, the lack of consistency and valuation of PRO data can create an ill-informed clinical environment, with different information about different concepts being used to make inconsistent decisions and to relay confusing information to patients. It also makes drug development difficult due to lack of clarity and consistency. The pharmaceutical industry may assume a precedent which may be unfounded (e.g., expectations of certain domains or endpoints being better valued by regulatory agencies based on previous PRO labelings); nevertheless, as feasible and as early as possible, pharmaceutical sponsors can and should engage with the FDA review divisions about the acceptability of their PRO endpoints prior to trial initiation. A further gap in PRO labeling from the pharmaceutical perspective is the under-representation of open-label or single-arm study designs.

#### 3.2.2 Clarity of patient-reported outcomes and applicability of patient-reported outcomes in labeling

**FIGURE 2:**
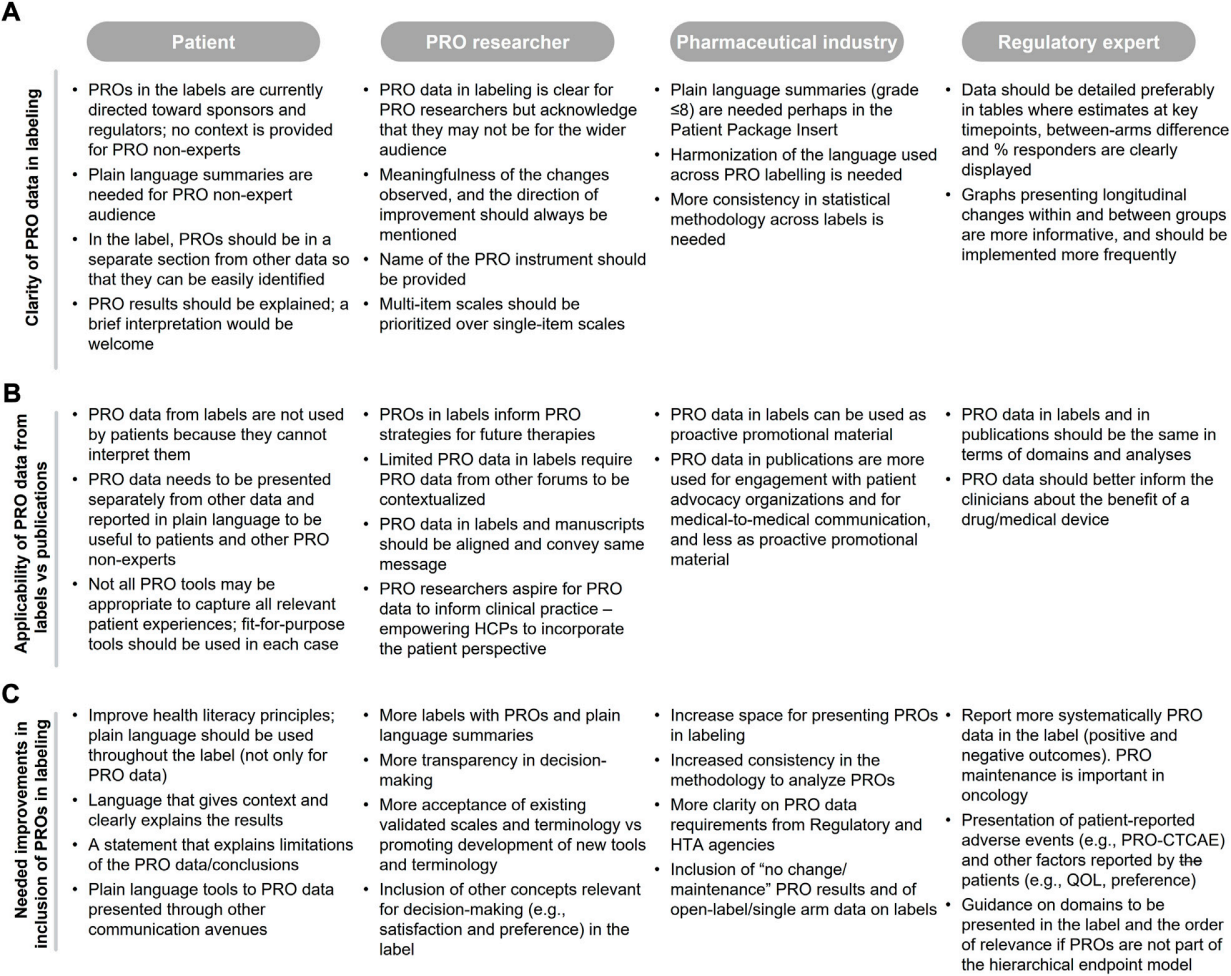
Authors feedback on **(A)** clarity of PRO data in labeling, **(B)** applicability of PRO data from labeling PRO data from publications, and **(C)** needed improvements in the context of PRO labeling.

From the patient and pharmaceutical industry perspectives, PRO data in labeling are not clear, and plain language summaries should be (but rarely are) included to aid interpretation for non-experts (Figure 2A). The lack of clarity means that the data is rarely used in clinical consultations (based on patients’ perspective), even though this is one of the goals of the collection of PRO data (Figure 2B). Even for experienced researchers and regulatory experts, it is important to present data in a way that facilitates interpretation of meaningfulness of the PRO findings. Specifically, clinical meaningfulness of the changes observed and the direction of improvement should be better specified in labels and should be consistent with other communications of PRO data, including publications (PRO researchers). More details on the methodology (pharmaceutical industry) and specificity of the data (regulatory expert) are also warranted. In addition, multi-item scales may be more informative than single item scales (PRO researchers) and should be prioritized where clear and easy to interpret (Figure 2A).

Authors considered the applicability of current PRO labeling as limited because they do not capture the full patient experience; only one or few of the concepts assessed in the registrational trials are captured in the label [e.g., labeling restricted to pain using the Brief Pain Inventory for abiraterone in the chemotherapy-naive setting with no reference to any Functional Assessment of Cancer Therapy—Prostate (FACT-P) domains such as prostate cancer symptoms, and impact of prostate cancer on social and family well-being, functional well-being, emotional well-being, physical well-being and quality of life]. All authors considered that when PRO instruments are fit for purpose and data are reliable, the label should include the same PRO endpoints that are communicated in other forums, such as publications (Figure 2B) with appropriate caveats, and that this information should be presented separately from other data to facilitate clarity.

Existing PRO labels are used, in part, to guide pharmaceutical industry and PRO researchers on the PRO strategy development for new drugs; although this information alone is rarely sufficient without understanding and stating the reasons why PROs were or were not included in the label (Figure 2B).

#### 3.2.3 Potential improvements in patient-reported outcomes in labeling

Numerous improvements were proposed by the authors, mainly related to the clarity and consistency of PRO data presentation (see Section 3.2.2), further guidance and transparency in the decision-making process, inclusion of non-traditional PRO data which is important for patient decision-making in clinical practice, and the inclusion of appropriately caveated PRO data from open-label and single arm studies where design constraints are relevant and justifiable (Figure 2C). These study designs are becoming more common in oncology to support accelerated approval (Kanapuru et al., 2017). Additional suggested changes are inclusion of findings that fail to show statistical significance but are clearly explained (opposed to assuming that absence of data indicates a negative finding/lack of data), and language that is more easily interpretable for communication by physicians to patients and caregivers. PROs that may not be eligible for labeling could still be endorsed by regulatory bodies and made available using platforms such as PPV; a pilot online platform launched by FDA in June 2020 to facilitate patients, caregivers and healthcare providers access to patient-reported treatment-related symptom data collected from oncology trials (FDA, 2022). At the time of the review, only one study was included in the PPV website (see Supplementary Material). Effort should also be made to accept and adapt existing validated PRO instruments in the interim rather than solely promoting development of new instruments which often take time for international validation and uptake and may not align with clinical trial schedules (PRO researchers; Figure 2C).

## 4 Discussion

Our findings suggest that despite the commitments from FDA and EMA to advance patient-focused drug development to capture the patient’s voice in clinical research, the role of PROs in regulatory labeling is still suboptimal. Even with the recent increase in the number of drugs with PRO labeling granted by FDA and EMA, the number of drugs with PRO data in the label, and the level of information provided in the label, underrepresents the available PRO evidence for oncology drugs. Unlike clinical endpoints such as overall survival for which all relevant information is generally provided, 22% of FDA and 57% of EMA labels do not provide any numerical estimates or refer to whether the PRO outcomes were statistically and/or clinically meaningful. Difficulty to interpret the PRO data in the labels has also been reported by others (Gnanasakthy et al., 2022).

Most PRO labeling relate to a single PRO concept, even when the registration trial assessed multiple ones. Inclusion of only selected PRO data may reflect a decision of the drug sponsor to submit partial data only or may result from a regulatory restriction; our analyses are unable to discern between these two possibilities. While it can be argued that not all PRO data may be sufficiently and scientifically robust, relevant, and interpretable to be included in the label, some of these data could also be made available on online platforms such as the FDA’s PPV, launched in June 2020 to communicate patient-reported treatment-related symptom data collected from oncology trials that are not included in the FDA label (FDA, 2022). However, since its creation, PPV provides data from only a single trial.

One of the factors that has limited PRO inclusion in labels is the unblinded nature of many oncology studies. Although there is evidence that the potential open-label bias for self-reported outcomes is much smaller than initially considered (Atkinson et al., 2017; Chakravarti et al., 2018; Mouillet et al., 2020; Efficace et al., 2022), and both FDA and EMA have shown willingness to include PRO findings from open-label studies in some oncology and non-oncology labels (Roydhouse et al., 2019), FDA has maintained that patients may provide biased reports of symptoms in trials that are either unblinded, or where study allocation could be revealed by differences in visible side effects between treatment arms (U.S. Department, 2009; Gnanasakthy et al., 2016). EMA is also concerned about potential bias in open label randomized studies but acknowledges that in certain cases the clinical evidence can only be obtained using this study design (EMA Committee for Medicinal Products for Human Use, 2019). Overall, 4 (44%) and 28 (49%) PROs in FDA and EMA labels, respectively, originated from open-label studies.

Another factor precluding inclusion of PRO in labels is failure to include PROs in the analysis hierarchy, often relegating them as exploratory endpoints. However, this does not preclude inclusion of PRO in labels even when the PRO endpoints are not included in the multiplicity hierarchy. PROs in 2 (22%) of the FDA labels were based on either exploratory endpoints or exploratory analyses of secondary endpoints. For EMA approvals, only two labels (sonidegib; vandetanib) specify the exploratory nature of the endpoint, overall the PRO labeling was based on exploratory endpoints only or with secondary endpoints in seven and two cases, respectively. In addition, one PRO labeling (blinatumomab) was based on *post-hoc* analyses of a secondary endpoint. Based on patients feedback, they consider that all endpoints and analyses should be prespecified in the clinical trial protocol. The low number of oncology drugs with PRO data in the label may also be due to the increasing number of oncology trials assessing more than one indication and the challenges to collect PROs in these trials. However, while this may be true in early phase trials, the majority of phase three trials focus on a single indication.

Our appraisal identified several gaps and areas for changes. Lack of plain language in the labeling, difficulty to discern from all other data in the label and use of complex graphs (Brundage et al., 2015), result in an underuse of any label data by clinicians and patients. While the lack of consistency across labels in the reported PRO concepts and analyses reflects the differences in the patient experience across and within oncology diseases, authors highlighted the need to identify core outcome sets and common data elements to be assessed across studies, and to gain agreement on analytical approaches to be taken for consistency. In addition, PROs in labeling often come from blinded comparative studies only and are biased towards positive findings (i.e., improvement). Data from other study types such as open label, single arm or patient preference studies may be informative to patients and healthcare providers, with the right caveats to account for potential biases.

Our study has several limitations. Our findings may not be generalizable to other therapeutic areas or diseases, particularly in those where PROs are often primary endpoints in clinical trials. The recent Eastern Research Group, Inc. review of FDA labels showed that PRO labeling did not exceed 17% of labels in any therapeutic area for new molecule entities approved by FDA between June 2017 and June 2020, and approved by the FDA Center for Drug Evaluation and Research or Center for Biologics Evaluation and Research by 5 February 2021 (ERG, 2019). These percentages are not fully consistent with those reported by Gnanasakthy et al. (2022). The authors observed an important difference in the proportion of new drugs approved by FDA between 2016 and 2020 with PRO labeling across therapeutic areas and in particular between PRO-dependent and PRO-independent therapeutic areas when PRO dependency is defined as diseases that rely on PRO assessments to derive or construct the primary or secondary endpoints for the evaluation of treatment benefit by regulators. The average proportion of labels for PRO-dependent diseases with PRO labeling was 50.0% vs. 9.7% for non-PRO-dependent diseases and 3.2% for oncology labels (Gnanasakthy et al., 2022). The 3.2% is lower than our findings (8.3%) probably because of the exclusion of biosimilars in the Gnanasakthy et al. study. Finally, the number of authors providing perspectives from the different standpoints is not equal across the four stakeholders. While three authors provided their perspectives from a pharmaceutical company and four from a PRO researcher, only one patient gave insights from a patient standpoint and one from a regulatory expert standpoint. The patient and regulator expert’s perspectives may not represent the insights of the full patient and regulators communities, respectively.

Our review does not inform on the number of unsuccessful attempts from sponsors in pursuing PRO labeling or reasons for failure. The Eastern Research Group, Inc.’s recent assessment of the use of PROs in FDA labeling shows that only 6% of oncology drugs for which PROs were included in the submission package received PRO labeling (ERG, 2019). This suggests that the low proportion of oncology drugs with PRO data in the label is not due to PROs being assessed in only few clinical trials. However, future research should investigate the proportion of clinical trials that include PRO collection, the number and variety of instruments included in novel clinical trials, and the proportion of regulatory agencies’ reviews that include thoughtful assessment of PROs evidence that may not have led to the incorporation of PRO evidence in drug labels. Our analysis does not provide information on whether PROs were considered in the risk-benefit assessment conducted by FDA or EMA, either. Finally, the current study focused only on PROs; we did not assess the inclusion of other clinical outcome assessments (i.e., clinician-reported, observer-reported or performance outcomes). In our analysis, we also did not identify any oncology drug providing data on caregiver burden despite oncology diseases having a negative impact on caregiver health-related quality of life (Rha et al., 2015).

A strength of this review is that it used a multidisciplinary approach where different stakeholders critically appraised the status quo in PRO labeling. However, future research should also analyze perspectives from treating clinicians and assess if they review and benefit from PRO label data.

The authors acknowledge the great advances incurred in the inclusion of patient voices in regulatory decisions. However, like others (ERG, 2019; Gilead and Regeneron, 2022; Gnanasakthy et al., 2022), we advocate for more inclusion of meaningful patient experience in those decisions.

## Data Availability

The original contributions presented in the study are included in the article/Supplementary Material, further inquiries can be directed to the corresponding author.
