# Supplementary material for: Patient-reported outcomes labeling for oncology drugs: Multidisciplinary perspectives on current status and future directions
Source: Front Pharmacol. 2022 Oct 17;13:1031992. doi: 10.3389/fphar.2022.1031992 (PMC9634749; doi:10.3389/fphar.2022.1031992)
Supplement: Supplementary file 1 [file DataSheet1.docx]

**Supplemen**t

**Table S1 Questions used to appraise the PRO labeling**

| 1. What do you see as the advantages and disadvantages of PRO data being included in drug labeling?  2. PRO data in drug labels is not consistent. A number of different signs, symptoms and impacts have been included in labeling, derived from a variety of different instruments implemented in different study designs. What do you see as the advantages and disadvantages of this?  3. Is the PRO data (text, graphs and tables) in drug labels informative and clear for you? Please provide examples.  4. How do/will you use PRO data in labels? Do/would you use PRO data in labels differently from PRO data in publications?  5. What improvements would you like to see in PRO labeling?  6. What other avenues are appropriate for presentation of PRO data? |
| --- |

**Table S2 FDA and EMA labels reviewed**

| **Drug** | **Indication with PRO (year*)** | **PRO type (pain, fatigue functioning...)** | **PRO instrument** |
| --- | --- | --- | --- |
| **FDA** |  |  |  |
| Abiraterone acetate | Chemo-naive prostate cancer (2016) | Pain (progression)  Opioid use | BPI-SF |
| Ceritinib | NSCLC (2014) | Breathlessness | Not specified |
| Crizotinib | NSCLC (2016) | Symptoms (dyspnea, cough, chest pain) | Not specified |
| Darolutamide | nmCRPC (2019) | Pain (progression)  Opioid use | Brief Pain Inventory-Short form (BPI-SF) |
| Fedranitib | Myelofibrosis (2019) | Symptoms (abdominal discomfort, pain under left ribs, night sweats, itching, bone/muscle pain and early satiety) | Myelofibrosis Symptom Assessment form (MFSAF) v2.0 diary |
| Pertuzumab (biosimilar) | Early HER2 positive breast cancer (2020) | Treatment administration preference | Preference questionnaire |
| Rituximab, hyaluronidase human (biosimilar) | First line DLBCL (2017) | Patient preference (revealed) | Preference questionnaire |
| Ruxolitinib | Primary Myelofibrosis (2017) | Symptoms (Fatigue) | modified Myelofibrosis Symptom Assessment Form (MFSAF) v2.0 diary  PROMIS® Fatigue |
| Trastuzumab hyaluronidase-oysk (biosimilar) | Breast cancer (2019) | Patient preference (revealed) | Preference questionnaire |
| **EMA** |  |  |  |
| Abemaciclib | Breast cancer (2018) | HRQoL | Not specified |
| Abiraterone acetate | Chemo-naive mCRPC (2017) | Pain (progression) | FACT-P  Instrument to assess pain was not reported |
| Abiraterone acetate | Post-chemo mCRPC (2011) | Pain (intensity) | BPI-SF |
| Afatinib | NSCLC (2013) | HRQoL  Functioning  Symptoms: cough, dyspnea and pain (progression) | EORTC QLQ-LC13  EORTC QLQ-C30 |
| Apalutamide | nmCRPC (2019) | HRQoL / functioning | FACT-P |
| Atezolizumab | NSCLC (2017) | Symptoms (SoB, cough, chest pain, arm/shoulder pain) | EORTC QLQ-LC13 |
| Atezolizumab | Breast cancer (2019) | HRQoL | EORTC QLQ-C30 |
| Azacytidine | AML with > 30% marrow blasts (2019) | HRQoL | EORTC QLQ-C30 |
| Binimetinib | Melanoma (2018) | HRQoL  Health status  Symptoms  Function | FACT-M  EORTC QLQ-C30  EQ-5D-5L |
| Blinatumomab | CD19 expressing B-cell precursor ALL (2018) | HRQoL  Symptoms  Functioning | EORTC QLQ-C30 |
| Brentuximab vedotin | Hodgkin lymphoma (2012) | HRQoL | QLQ-C30 |
| Brentuximab vedotin | Cutaneous T cell leukemia (2018) | HRQoL  Health status | FACT-G  EQ-5D |
| Cabazitaxel | Post chemo mCRPC (2011) | Pain | McGill Pain Questionnaire (MPQ) |
| Cabozantinib | Hepatocellular carcinoma (2019) | HRQoL | EORTC QLQ-C30 |
| Carfilzomib | Multiple myeloma (2015) | HRQoL | EORTC QLQ-C30 |
| Ceritinib | NSCLC (2017) | Symptoms of lung cancer (cough, pain, dyspnea)  HRQoL  Health status | EORTC QLQ-LC13  LCSS  EORTC QLQ-C30  EQ-5D |
| Cobimetinib | Melanoma (2015) | HRQoL  Symptoms (appetite loss, constipation, nausea and vomiting, dyspnea, pain, fatigue) | EORTC QLQ-C30 |
| Crizotinib | NSCLC (2016) | Symptoms (chest pain, cough, dyspnea)  HRQoL | EORTC QLQ-C30  EORTC QLQ-LC13 |
| Darolutamide | nmCRPC (2020) | Pain | BPI-SF |
| Denosumab (biosimilar) | Giant Cell Tumor of Bone (2011) | Pain (progression)  Analgesic consumption | BPI-SF  Analgesic use graded on a seven-point scale |
| Durvalumab | NSCLC (2018) | HRQoL  Function  Symptoms | EORTC QLQ-C30  EORTC QLQ-LC13 |
| Encorafenib | Melanoma (2018) | HRQoL  Function  Symptoms  Health state | FACT-M  EORTC QLQ-C30  EQ-5D-5L |
| Enzalutamide | Chemo-naive mCRPC  (2013) | HRQoL  Function | FACT-P |
| Idelalisib | CLL (2014) | Function  Health status | FACT-Leu  EQ-5D |
| Inotuzumab ozogamicin | pre B-ALL (2017) | HRQoL  Functioning  Health status  Symptoms | EORTC QLQ-C30  EQ-5D |
| Ixazomib | Multiple myeloma (2016) | HRQoL | EORTC QLQ-C30  EORTC QLQ-MY20 |
| Lutetium (177Lu) oxodotreotide | Neuroendocrine Tumors (2019) | HRQoL | EORTC QLQ-C30  EORTC QLQ-GINET21 |
| Nintedanib | NSCLC (2015) | Symptom (cough, dyspnoea, pain, diarrhea)  HRQoL | Not specified |
| Niraparib | Ovarian, fallopian tube, or primary peritoneal cancer (2017) | HRQoL | EQ-5D  FACT-O |
| Nivolumab | Head and neck cancer (2017) | Functioning  Health status  Symptoms | EORTC QLQ-C30  EORTC QLQ-H&N35  EQ-5D |
| Nivolumab | NSCLC (2015) | Symptom  Health status | LCSS  EQ-5D |
| Nivolumab | Renal cell carcinoma (2016) | HRQoL  Symptoms | EQ-5D  FKSI-DRS |
| Nivolumab | Melanoma (2015) | HRQoL  Health status | EORTC QLQ-C30  EQ-5D |
| Obinutuzumab | CLL (2014) | HRQoL  Fatigue | EORTC QLQ-CLL-16  EORTC QLQ-C30 |
| Obinutuzumab | Follicular lymphoma (2016) | HRQoL  Health status | FACT-Lym  EQ-5D |
| Obinutuzumab | Tx naive follicular lymphoma (2014) | HRQoL  Health status  Symptoms | FACT-Lym  EQ-5D |
| Olaparib | Ovarian, fallopian tube, or primary peritoneal cancer (2015) | HRQoL  Symptoms | FACT-O  NCCN-FACT FOSI-18 |
| Osimertinib | 1L NSCLC (2017) | HRQoL  Symptoms | EORTC QLQ-C30  EORTC QLQ-LC13 |
| Osimertinib | RR NSCLC (2017) | Symptoms  HRQoL  Functioning | EORTC QLQ-C30  EORTC QLQ-LC13 |
| Padeliporfin* | Prostatic cancer (2017) | Erectile function  Urinary symptoms | IIEF-15  I-PSS |
| Palbociclib | Breast cancer (2016) | Pain  Symptoms | EORTC QLQ-C30  EORTC QLQ-BR23 |
| Pazopanib | Renal cell carcinoma (2010) | HRQoL  Health status | EORTC QLQ-C30  EQ-5D |
| Pembrolizumab | Bladder cancer (2017) | HRQoL | EORTC QLQ-C30 |
| Pertuzumab | Metastatic breast cancer (2015) | HRQoL | FACT-B |
| Pertuzumab | Early stage breast cancer (2018) | HRQoL  Functioning  Symptoms | EORTC QLQ-C30  EORTC QLQ-BR23 |
| Radium Ra 223 dichloride | Bone metastases due to mCRPC (2013) | HRQoL  Functioning  Health status  Pain (relief) | FACT-P  EQ-5D  Pain relief assessment method not mentioned in the label |
| Ramucirumab | NSCLC (2016) | HRQoL | Lung Cancer Symptom Scale (LCSS) |
| Ramucirumab | Colorectal cancer (2016) | HRQoL | EORTC QLQ-C30 |
| Ribociclib | Breast cancer (2017) | HRQoL | Not reported |
| Sonidegib | Basal cell Carcinoma (2015) | Functioning  Symptoms | EORTC QLQ-C30  EORTC QLQ-H&N35 |
| Tisagenlecleucel | ALL (2018) | HRQoL  Health status | EQ-5D  PedsQL™ |
| Trastuzumab emtansine | Breast cancer (2013) | Symptoms (progression) | FACT-B |
| Vandetanib | Thyroid cancer (2012) | Symptoms (Pain; Stool frequency)  Opioid analgesic use | BPI  Stool frequency measurement not specified  Opioid analgesic use |

*Year of approval for the indication with the PRO labeling. Abbreviations: ALL: acute lymphoblastic leukemia; AML: acute myeloid leukemia; BPI-SF: Brief Pain Inventory-Short form; cGVHD Symptom Scale: Lee Chronic Graft-versus-Host Disease Symptom Scale; CLL: chronic lymphocytic leukemia; DLBCL: diffuse large b cell lymphoma; EMA: European Medicines Agency; EORTC QLQ-BR23: breast cancer module of the EORTC QLQ-C30; EORTC QLQ-C30: European Organisation for Research and Treatment of Cancer quality of life questionnaire core; EORTC QLQ-CLL17: chronic lymphocytic leukemia module of EORTC QLQ-C30; EORTC QLQ-GINET21: gastrointestinal neuroendocrine tumors module of the EORTC QLQ-C30; EORTC QLQ-H&N35: EORTC QLQ-H&N35: head and neck cancer module of the EORTC QLQ-C30; EORTC QLQ-LC13: lung cancer module of the EORTC QLQ-C30; EORTC QLQ-MY20: EORTC QLQ-MY20: myeloma module of the EORTC QLQ-C30; EQ-5D: EuroQoL 5 dimension; FACT-B: FACT-B: breast cancer module of the FACT-G; FACT-G: Functional Assessment of Cancer Therapy – General; FACT-Leu: FACT-Leu: leukemia module of the FACT-G; FACT-Lym: lymphoma module of the FACT-G; FACT-M: myeloma module of the FACT-G; FACT-O: ovarian cancer module of the FACT-G; FACT-P: prostate cancer module of the FACT-G; FDA: Food and Drug Administration; FKSI-DRS: FKSI-DRS: Functional Assessment of Cancer Therapy – Kidney Symptom Index; HRQoL: health-related quality of life; IIEF: International Index of Erectile Function; I-PSS: International Prostate Symptom Score; LCSS: Lung Cancer Symptom Scale; mCRPC: metastatic castration-resistant prostate cancer; MFSAF: Myelofibrosis Symptom Assessment form; MPQ : McGill Pain Questionnaire; NCCN-FACT FOSI-18: FACT-G Ovarian Symptom Index 18; nmCRPC: non-metastatic castration-resistant prostate cancer; NRS: numeric rating scale; NSCLC: non-small cell lung cancer; PedsQL™: Pediatric Quality of Life Inventory; PRO: patient-reported outcomes; PROMIS: Patient-Reported Outcomes Measurement Information System; QoL: quality of life; RR: relapsed or refractory; Tx: treatment; VAS: visual analogue scale.

**Table S3 PRO instruments mentioned in PRO labeling**

|  | **FDA** | **EMA** |
| --- | --- | --- |
| Generic PRO instruments |  |  |
| BPI-SF | 2 |  |
| EORTC QLQ-C30 | - | 2 |
| EQ-5D | - | 16 |
| IIEF | - | 1 |
| I-PSS | - | 1 |
| FACT-G | - | 1 |
| Pain relief assessment no further specified | - | 1 |
| PedsQL | - | 1 |
| Preference questionnaire | 3 | - |
| PROMIS Fatigue | 1 | - |
| Stool frequency measurement not specified | - | 1 |
| Not specified | 2 | 2 |
| Disease-specific PRO instruments |  |  |
| EORTC QLQ-BR23 | - | 2 |
| EORTC QLQ-CLL16 | - | 1 |
| EORTC QLQ-GINET21 | - | 1 |
| EORTC QLQ-H&N35 | - | 2 |
| EORTC QLQ-LC13 | - | 7 |
| EORTC QLQ-MY20 | - | 1 |
| FACT-B | - | 2 |
| FACT-Leu | - | 1 |
| FACT-Lym | - | 2 |
| FACT-M | - | 2 |
| FACT-O | - | 2 |
| FACT-P | - | 3 |
| FACT FOSI-18 | - | 1 |
| FKSI-DRS | - | 1 |
| LCSS | - | 4 |
| MPQ | - | 1 |
| MFSAF v2.0 diary | 2 | - |

Abbreviations: BPI-SF: Brief Pain Inventory-Short Form; cGvHD: chronic graft-versus-host disease; cGvHD: chronic graft-versus-host disease symptom scale; EORTC QLQ-30: European Organisation for Research and Treatment of Cancer quality of life questionnaire core; EORTC QLQ-BR23: breast cancer module of the EORTC QLQ-C30; EORTC QLQ-CLL17: chronic lymphocytic leukemia module of EORTC QLQ-C30; EORTC QLQ-GINET21: gastrointestinal neuroendocrine tumors module of the EORTC QLQ-C30; EORTC QLQ-H&N35: head and neck cancer module of the EORTC QLQ-C30; EORTC QLQ-LC13: lung cancer module of the EORTC QLQ-C30; EORTC QLQ-MY20: myeloma module of the EORTC QLQ-C30; EQ-5D: EuroQoL 5 dimension; FACT FOSI-18: FACT-G Ovarian Symptom Index 18; FACT-B: breast cancer module of the FACT-G; FACT-G: Functional Assessment of Cancer Therapy – General; FACT-Leu: leukemia module of the FACT-G; FACT-Lym: lymphoma module of the FACT-G; FACT-M: myeloma module of the FACT-G; FACT-O: ovarian cancer module of the FACT-G; FACT-P: prostate cancer module of the FACT-G; FKSI-DRS: Functional Assessment of Cancer Therapy – Kidney Symptom Index; IIEF: International Index of Erectile Function; I-PSS: International Prostate Symptom Score; LCSS: Lung Cancer Symptom Scale; MFSAF: Myelofibrosis Symptom Assessment form; MPQ: McGill Pain Questionnaire; NRS: numeric rating scale; PedsQL: Pediatric Quality of Life Inventory; PROMIS: Patient-Reported Outcomes Measurement Information System; VAS: visual analogue scale. For further information, please check: <https://qol.eortc.org/> (for EORTC tools), <https://www.facit.org/> (for all FACIT / FACT- tools) and <https://euroqol.org/> (for EQ-5D).

**Review of the PPV website**

Since June 2020 when the Patient Project Voice (PPV) website was created, only data related to a single drug (osimertinib; Tagrisso®) in one clinical study (AURA3; NCT02151981) in locally advanced or metastatic non-small cell lung carcinoma has been uploaded. The PPV website provides an overview of the study design and a summary of demographics. The website also provides a table with the data related to the first attribute of the PRO-CTCAE items collected in the study (https://www.fda.gov/about-fda/project-patient-voice/aura3). The study is supplemented with a legend explaining how to interpret each column of the table and each row is linked to a separate tab where the data for each PRO-CTCAE symptom is further discussed.

**Definitions used in the manuscript**

Exploratory endpoints are defined by FDA as 1) endpoints assessing events that are expected to occur too infrequently to show a treatment effect or 2) endpoints that for other reasons are thought to be less likely to show an effect but are included to explore new hypotheses.

(Drug) label relates to both the US Prescribing Information and the European summary of product characteristics.

Post-hoc analyses indicate that the analyses were conducted once the data from the study had already been analyzed. These analyses are not prespecified in the statistical analysis plan, i.e., the document that is developed prior to data analysis and, if blinded, prior to unblinding the study. This document specifies all analyses that will be conducted.

Registrational trial is the clinical study conducted to support regulatory approval of a therapy.
